# Chicago Pathological Society

**Published:** 1884-12

**Authors:** 


CHICAGO PATHOLOGICAL SOCIETY. *April 14, 1884. Regular Meeting.*

The Society was called to order by the President, Dr. Angear.

Dr. Campbell was elected Secretary, *pro tem.*

After opening the meeting the President was summoned away, and called Dr. Lyman to the chair.

Dr. P. C. Jensen, 128 Milwaukee avenue, Chicago, was unanimously elected to membership.

Dr. A. E. Hoadly was proposed for membership by Drs. Skeer and Lyman. It was then moved and seconded that as regarding election of delegates to the American Medical Association, any member wishing to act as such should call on the Secretary for suitable credentials. Carried.

Dr. J. D. Skeer then reported an interesting case of impacted fracture of the femur, with the following history: Mrs. C., aged eighty years, of fair constitution, but, on account of advanced age, of relaxed muscular fiber and exhausted nervous system. In passing from one room to another she tripped on a rug and fell to the floor. The force of the fall must have been expended near the *trochanter major.* Found her lying on the floor on the back, the right foot everted and the limb shortened; she was almost overcome by the shock, and in great pain at hip joint. The femur would rotate all the way up to the socket. There was no crepitus, and the Doctor had no hesitation in pronouncing it a case of impacted fracture of the femur. Notwithstanding considerable deformity, best seen by examining the specimen, he made no attempt to reduce the fracture, and no effort should be made to reduce an impacted fracture of the neck of the femur; on the contrary, the indications are to preserve the impaction; but extension by weight and pulley, to overcome muscular contraction, may be necessary to prevent displacement. In this case he made no extension, applied no splints, placed the limb on an inclined plane, made of pillows, and directed hot water dressings; giving anodynes to relieve pain and encourage reaction. Tonics and stimulants were given as needed and required, and good nursing maintained. The patient lingered in a low condition for fourteen weeks, when she died from exhaustion.

This case is interesting on account of the age of the patient, the apparently trivial cause of the accident, the length of time she lived in her exhausted condition, the extent of the fracture and the amount of repair.

One notices in the specimen a fracture on the posterior aspect of the bone, extending from the base of the *trochanter major* to the *trochanter minor*; both trochanters, including the intervening fragments of bone, being rotated outward to an angle that would be equal to about the fifth of a circle. Another fracture in front, along the spiral line, at the base of the neck; the back part of the neck being crushed into the fissure between the fragment and the body of the bone—the neck being at a right angle to the axis of the femur. We find the bones pretty firmly united in their abnormal position by osseous union. It may not be necessary to observe that this fracture is outside of the capsule.

*Case II. Stricture of the Sigmoid Flexure of the Colon. Reported by Dr. Skeer.*—Mr. B., aged 62 years, born in Scotland, has been a resident of this country for 33 years, a carpenter by trade. Never had much sickness. Brother died at the age of 59 of cancer of the stomach, as demonstrated by post-mortem examination. On the 28th day of February last he complained of constipation, and took a dose of castor oil, which did not operate. The next day called Dr. Griswold, who gave him mass. hydrarg, and followed it with compound licorice powder, which produced a free and satisfactory operation.

Dr. G. did not see him again until March 10. In the meantime patient had taken from a neighboring physician drop doses of croton-oil every three hours until six drops were taken. The oil excited violent retching and vomiting, without operating on the bowels.

Dr. Earle was called in consultation, and advised the administration of pepsine and bismuth to allay the vomiting, and large warm water injections to move the bowels, which were continued for five or six days, without any movement of the bowels.

Dr. Skeer saw him for the first time on March 15th. He was almost free from pain, abdomen enormously though uniformly distended, no pain on pressure, not much fever nor acceleration of the pulse, and some sickness of the stomach. Ether was then given, and the rectum explored; a hard mass was felt projecting down between the rectum and the bladder. On passing the hand into the rectum the sense of touch conveyed the impression as though the hand was being inserted into the bladder. Prolapsus of the bowel was diagnosticated, and perhaps an organic stricture in the sigmoid flexure of the colon.

On the 10th of March, Drs. Hoadley and Graham were added to the council. Preparation was made to open the abdomen, if necessary, to restore the prolapsed bowel. Patient being etherized, Dr. Hoadley passed his hand to the sigmoid flexure of the colon, the prolapsed bowel disappearing before it, and he could find no stricture; it was thought best to defer further interference and await developments. The following day the patient had a free movement of the bowels, and passed off considerable gas. The distention of the abdomen, however, continued as before; liquid nourishment and supporting treatment were given, but no anodynes required. The patient grew gradually worse, without much change and without any further movement of the bowels; died on the first day of April, just one month from the commencement of the obstruction.

Post-mortem examination twenty-four hours after death. Only the contents of the abdominal cavity were examined; the peritoneum inflamed, covered with lymph.

Removed sigmoid flexure, and about its center was formed an organic stricture which almost closed its caliber. Above this the bowel was very much distended, and contained a large quantity of hardened fæces, forming a pouch which had been crowded down into the pelvis between the rectum and the bladder. Above and just at the seat of the stricture were two large perforating ulcers and an orange seed.

The immediate cause of death was peritoneal inflammation following perforation.

The Society then proceeded to discuss the case above reported.

Dr. Lyman remarked that it was usual in cases of obstruction low down, to find but little or no pain, but when located near the stomach, pain was great.

Dr. C. J. Lewis spoke of a recent case of chronic bronchitis where he was able to obtain a post-morten. He failed on examination of the lungs to find a single normal cell. The upper third of one lung was entirely absorbed, and a large cavity in the other; general pleuritic adhesions. At no time was the pulse above 100. Average 80 to 84. There was tubercle present in quantity.

Dr. Lyman saw the case a short time before death, and thought the general symptoms were either of cavities by ulceration or of enlarged bronchial tubes. The age was fifty-six.

Calling Dr. Skeer to the chair, Dr. Lyman then reported a post-mortem case.

The man, age forty-five, seaman, owned his bark, and was, in October, 1881, out on the lake during the memorable gale; had never been so well since. During winter of 1883 his character changed, he became despondent, worried and fretted about his ship; on starting on his voyage he soon acted strangely—hilarious, talkative, and quarrelsome—was taken ashore and home; Dr. Lyman summoned. His pulse was rapid, face suffused, and had not slept for a long time. He was full of moneymaking projects. Under treatment some improvement was made during the autumn, but in winter his despondency returned with suicidal impulses; timid upon going out alone. He realized that he had insane feelings, and was, by his own wish, sent to the asylum. Made his will. For several months afterward was quiet and contented, but dared not leave, and still had a tendency to despondency. Retired one night as usual, in good condition, and was found dead in the morning, without any sign of a struggle, violence or poison.

Post-mortem revealed, on exposing the brain, great dryness of its surface. To the eye, its color, texture, and appearance were natural. The *medulla oblongata* and *pons varolii* were softened, hence supposed the disease to lie in the blood-vessels of the brain, of such character as to cause death of the pons and medulla, so that breathing could no longer be carried on. No opportunity was afforded for making a microscopic examination. Dr. Lyman thought the case was one of so-called "circular insanity," beginning with melancholy, then exhilaration, followed by an intermission of nearly a sane condition, then melancholia again.

These phases may occupy a few days, or as many weeks, in passing, but may change as seasons change.

Dr. Campbell then inquired if the condition was caused by the shock of fright during the severe storm, and Dr. Lyman replied that he thought so, and that the man had considerable predisposition to cerebral excitement.

Dr. Haven was reminded of a case seen in Wisconsin, of "circular insanity," where the changes varied with the seasons; all right, or nearly so, during the summer, but insane during the cold weather.

Dr. Lyman stated that the intervals may be long between the phases, then again the patient may go to bed and asleep in one phase, and awake in another. He then stated that in the case he had reported there was no trace in the family history, either of specific disease or injury of any kind.

Present, about fifteen members and several visitors.

The Society, on motion, adjourned.

J. H. TEBBETTS, *Secretary.*

---

*May 12, 1884, Annual Meeting.*—The Society was called to order by the President, Dr. Angear.

The minutes of the last meeting were read and approved.

It being the annual meeting, the first business of the evening was the election of officers for the ensuing year. According to former custom, an informal ballot was taken for President, Dr. Angear receiving three votes, Dr. E. L. Holmes thiee, Dr. J. D. Skeer two, and a number of scattering votes.

On formal ballot. Dr. J. J. M. Angear received a majority of the votes and was declared elected.

Dr. E. L. Holmes was elected Vice-President; Dr. J. H. Tebbetts, Secretary and Treasurer; Drs. J. D. Skeer and H. M. Lyman, Censors.

The Secretary then read his annual report, which was afterward approved and accepted.

On motion of Dr. Lyman, the Society passed the Secretary a vote of thanks for said report, and requested its publication in the CHICAGO MEDICAL JOURNAL AND EXAMINER.

The Treasurer's report was then read and accepted; after which, the President, Dr. Angear, in a few remarks thanked the Society for his reëlection, bespeaking his interest in the Society, both in the past and in the future. He hoped the general interest of the members might be increased in some way; perhaps by contriving a few social meetings, followed by a supper, etc.; also, that dues could well be increased; that this Society had a place here in Chicago and was formed to grow; that outside members of the profession had little idea of the quality or quantity of its work, and did not realize how much they were missing in not becoming active members at once. Thought, also, the Secretary's suggestion, of a year ago, a good one: that of getting our constitution and by-laws printed, together with a revised list of membership. In conclusion, considered our present location a good one, and that the Society should settle down therein, and feel more at home.

It was then moved by Dr. Holmes that the President be thanked for his speech. Carried.

Dr. A. E. Hoadley was then elected to membership unanimously.

Dr. E. A. Baldwin, 84 Park avenue, was proposed for membership by Drs. Holmes and Dobbin.

The Society then listened with close attention to a verbal essay, by Dr. Lyman, upon "Atmospheric Dust in Relation to Disease;" and although a full abstract would be very valuable and interesting, a few notes only were recorded.

All are of course familiar with the fact that the atmosphere may become unfit for respiration, and that minute particles may be of material, palpable character, and are generally liable to produce disorders of the respiratory tract; hence, this ground does not need to be passed over; and, though familiar, is really not as yet definite.

All are also familiar with the injurious effects caused in certain arts and trades by flying particles of irritating matter,—from grindstones, emery wheels, flying cotton dust, grain dust, etc., all of which cause serious pharyngeal trouble. Again, all are acquainted with certain forms of asthma, caused by the atmosphere being charged with pollen of plants, grasses, etc., at certain times in the summer. A friend goes to Northern Michigan, every summer, to obtain relief from asthma, hay fever, and is there very comfortable indeed, except when a strong southerly wind blows for several hours, laden with dust or pollen; then is uncomfortable while wind remains in that quarter.

It may not be generally known that there are, besides these varieties of dust, dust from other sources, continually diffused; and first may be mentioned cosmic dust, which enters our atmosphere from space, and some of which passes back again, but much of it is constantly falling. A German scientist estimates the amount falling upon the earth at one half million tons annually, which is really a large amount, and perfectly appreciable when sought for.

The Challenger expedition found, in deep-sea soundings in the Pacific, little masses of pumice from volcanic sources; also, little microscopic spherules, perfectly round like shot, and as uniform in size and shape; these were composed of meteoric iron. When a meteor falls, the friction is so great, that the outer surface of the iron mass becomes broken off by heat scintillations, and these, in form of spherules, gradually settle, widely diffused, all over the surface of the globe. The same scientist found, on the surface of snow and ice in Greenland, this same deposit.

In addition to meteoric dust, there is terrestrial dust.

In California and Oregon there is found, at times, dust from the high interior plains of China, from across the Pacific; fine alluvial soil, in motion by the wind. Dust from the Desert of Sahara rains down on the Madeira, Canary, and Cape Verde Islands. Dust of the Pampas (Darwin) settles on the east side of the South Atlantic; also, dust from the high, arid plains of Utah and Colorado, is thrown up by the wind and blown great distances. Volcanic dust is transported to the highest altitude of any terrestrial dust. Whymper, when ascending Mt. Chimborazo, South America, observed an eruption on Mt. Cotopaxi, early in the morning, sixty-five miles to the northeast. A column of black smoke arose twenty thousand feet above the cone, in less than two minutes; it there reached an air-current going towards the Pacific Ocean, then a northerly current to the south, then came towards them, a circuit of over one hundred miles. Very fine color effects were produced by the dust hiding and intercepting the sun's rays; green, then lurid purple, unlike anything ever seen, and frightful in appearance; the dust blackened the snow and penetrated clothing and instrument cases.

The gorgeous sunrises and sunsets of last winter, were connected with and caused by volcanic dust.

The volcanic eruption in the Straits of Sunda, last October, was the greatest ever known, and its disturbance was felt all over the world, and probably had some effect upon the world's health.

Dr. Lyman then gave a fine and vivid description of the eruption. There is no known measurement of the amount of dust fallen; the sky completely darkened; vessels, many miles away, lighted binnacle and other lights at midday; a fearful rain of mud; the islands were covered feet deep with white volcanic dust, like snow; the sea surface dense with square miles of floating pumice, which finally becomes water-logged, its cellules filling, and then sinks.

Then arose the important question whether such an admixture of dust in the atmosphere may not be a cause of disease. It would be a difficult thing to prove, as there must be shown close connection between presence of the dust in quantity and any considerable outbreak of disease.

Dr. Lyman began to observe, about Jan. 1, a considerable number of cases of inflammation of the posterior nares and larynx, quite different from any ever seen before by him. The individual seemed in otherwise perfect health, no febrile movement; but suddenly there came on dryness of the pharynx and sensations analogous to local congestion; he observed this before his attention had been called to the dust by the scientific journals.

During ordinary weather there are no great changes between the lower and higher strata of the atmosphere; then, when the first cold wave ("cold snap") came, there was a down-pour of the upper atmosphere upon the lowest levels. This produces disturbances, high winds, and other like phenomena, and Dr. Lyman thought these little crystalline bodies thus suddenly descending subside slowly and drift like smoke.

Tyndall (" Tyndall on Dust ")observed that the atmosphere is always charged with dust, giving the sky its deep blue color by reflecting the blue rays of light and transmitting all the others. A close box is filled with smoke, with one aperature admitting a single ray of light. In this ray, dust is shown as blue; a heated bar held in this ray darkens the ray entirely, showing that the dust is cleared from that portion of the space. Tyndall at first thought the bar burned the dust, but afterward proved that radiant force repels all floating particles in the atmosphere. Still more powerful for this purpose is electricity; and after a severe thunder storm the air becomes, on the next day, wonderfully clear and pure, which is due to the electricity, and not to wind nor rain.

Dr. Lyman would now like to inquire of members if any noticed peculiar conditions of the respiratory organs during the past winter.

Dr. E. L. Holmes rose to inquire whether the action of seawater would not destroy all cosmic or meteoric dust in a short time, and he was answered that the material was so nearly chemically pure it oxidized but slowly, and the supply was constantly being recruited.

Dr. Robison stated that he had treated a great many cases of hyperæmic catarrh the past winter at the dispensary, and that there had really been a remarkable increase of these cases, while attendance had not perceptibly increased, and this condition is still present and attracts some attention.

Dr. Norman Bridge noticed during last winter, in private practice, a considerable increase of throat cases.

Dr. Lyman then stated that this volcanic dust had not yet all settled, and that with the hand shading the eyes, one can still see a hazy band on each side of the sun, even on a clear day.

Dr. Bishop expressed his interest in the subject and thought many individuals especially susceptible to this dust. Pollen arises to about 150 feet, and there are many with personal idiosyncrasies. Farmers seem to be less liable to hay fever than clergymen, lawyers and physicians; some individuals will have attacks of all the forms of asthma, others of one only.

Dr. Angear thought it evident that there must be more than the mechanical effect upon the lung tissue by the dust or pollen. Ordinary dust that can be coughed up is not necessarily injurious; irritating particles not so readily coughed up are injurious.

One observer noticed that dust from America was floating in the air in Africa, and vice-versa.

Dust may have originated in some morbid matter or fluid, and convey that material and contaminate the body. Dust may have, perhaps, some chemical influence unseen microscopically. A test should be employed, using large quantities.

Dr. E. L. Holmes stated that in powdered ipecac the drug can be measured, but not the individual idiosyncrasy.

Dr. Twining inquired what proportion cosmic or volcanic dust bore to the ordinary dust in the air, and Dr. Lyman replied that the proportion was very variable; there was no constant ratio.

Dr. Bishop inquired of Dr. Lyman whether he had had more throat cases during the fall than in the last two months; and Dr. L. replied, not particularly, but in character were peculiar.

Present, about sixteen members.

The Society, on motion, adjourned.

Park Institute. J. H. TEBBETTS, *Secretary.*

*Secretary's Annual Report for Year Ending April, 1884.—Meetings.*—During the past year the Society has held twelve regular meetings, at several different places. The first meeting of the year, the annual meeting, was held at 533 W. Adams street, May 14, 1883, and the last, April 14, 1884, at the Park Institute. The meetings from June to November, inclusive, were held in the new building of the College of Physicians and Surgeons. This location not proving sufficiently central and convenient for a majority of the members, the Society next held its meetings, during December and January, in the parlor of the Washingtonian Home; but in February, being unable to obtain this room any longer, found comfortable quarters at 105 S. Ashland avenue, in the Park Institute, where meetings have since been regularly held, making three months' time at the present location.

*Members.*—One hundred and sixty members have attended during the year, an average of about thirteen at each meeting. Thirteen ladies and gentlemen were proposed for membership during the year, all of whom were unanimously elected, and have proved active and valuable members.

*Postals.*—There have been printed and mailed to members, fourteen hundred and thirty-seven postals, giving time and place of meeting and order of exercises.

*Work of the Year.*—The following literary and scientific work has been accomplished by the Society during the year: Thirteen papers, seven detailed reports of particularly interesting cases, three announced discussions, and were entitled as follows:

1. Paper. Dr. G. Frank Lydston, "The Treatment of Varicocele."

2. Paper. Dr. E. P. Murdock, "The Immediate Operation for Repair of Lacerated Cervix."

3. Paper. Dr. John A. Robison, " Myxoedema."

4. Case Reports. Dr. E. R. Bennett, *a*, "Lumbago," *b*, " Peculiar Case of Syphilis."

5. Paper. Dr. D. R. Brower, " Chorea."

6. Case Report. Dr. E. P. Murdock, " Infantile Tetanus."

7. Paper. Dr. William A. Walker, " Operation of Lithotomy."

8. Paper. Dr. Chas. Warrington Earle, " Cephalhæmatoma of the New-born."

9. Paper. Dr. Earle, " Disease of the Spleen and Report of a Fatal Case."

10. Discussion. " Sponge Grafting," Drs. Angear, Newton, Tagert and Patton.

11. Paper. Dr. H. M. Lyman, " Apoplexy."

12. Paper. Dr. G. Frank Lydston, " Some of the Fallacies of Modern Sanitation, with Especial Reference to the Sewage System in Relation to Zymotic Disease."

13. Paper. Dr. R. J. Curtiss, " The Disposal of Chicago Sewage."

14. Paper. Dr. J. D. Skeer, " Malignant Intestinal Obstruction."

15. Discussion, " Rectal Feeding," Drs. Robison, Van Buren, Skeer, Lyman, Angear

16. Paper. Dr. J. J. M. Angear, " Expectant Treatment."

17. Paper. Dr. E. L. Holmes, " Melanotic Sarcoma of the Eye, with Reports of Cases."

18. Discussion. "Therapeutics of Malarial Disease." Drs. Holmes, Avery, Haven, Root, Lagorio, Bucher, Angear.

19. Case Report, with specimen. Dr. E. P. Murdock, " Monstrosities."

20. Case Reports. Dr. J. D. Skeer, (*a*) " Impacted Fracture of the Neck of the Femur;" (*b*) "Case of Stricture of the Sigmoid Flexure of Colon."

21. Case Report. Dr. H. M. Lyman, "Circular Insanity."

*Suggestions.*—It is sometimes a matter of some difficulty to obtain papers or case reports from members for our meetings. Many are not active members, though attending occasionally. Nearly all are in active practice, are frequently obtaining post-mortems, and with but a minimum amount of trouble and time, and a trifle of enthusiasm, might afford the Society much valuable matter for discussion and general interest. Verbal reports from the more busy members would be highly appreciated, and the usefulness of the Society much increased. The general work of the year has been of a higher standard than before, and is getting more nearly upon a strictly pathological basis—all matters of medical, surgical and pathological interest still receiving equal attention, the majority of our members being busy in general practice. It would seem desirable that the present location be permanently occupied, as frequent changes reduce our attendance, and no better hall is at present obtainable in as central a location.

Respectfully submitted,

J. H. TEBBETTS, *Secretary*.

---

*Treasurer's Report for the Year Ending April, 1884.*—

DR.

| | |
|---|---|
| To balance on hand May 14, 1883 | $ 4 98 |
| To annual dues collected | 60 00 |
| | $64 98 |

CR.

| | |
|---|---|
| By bills paid, hall rent, stationery, printing, etc | $41 01 |
| By cash on hand | 23 97 |
| | $64 98 |

J. H. TEBBETTS, *Treasurer*.

*June 9, 1884—Park Institute.*—The Society was called to order by the President, Dr. Angear.

The minutes of the last meeting were read and approved. Dr. E. A. Baldwin, 84 Park Avenue, Chicago, was unanimously elected to membership.

The Society then listened to the reading of a paper by Dr. G. Frank Lydston, entitled "The Prophylaxis of Puerperal Disease," an abstract of which is here omitted, as the paper is about to be printed in full.

The paper then being before the Society for discussion, Dr. J. D. Skeer thought it well to remember that parturition was a natural process, and fully agreed with the author of the paper that "let alone" treatment gave the best results ; he would, of course, use disinfectants occasionally; considered that the danger from septic absorption to be much less than we have supposed.

Dr. Twining approved of the suggestion of guarding the patient's nervous system from shock and weariness, and of discriminating between true puerperal fever and the fever of nervous excitement merely. Have always had satisfaction in the use of vaginal injections of warm water, adding by preference chlorinated soda, but sometimes carbolic acid.

Dr. H. M. Lyman was interested in the paper, and thought it a timely one, much needed at the present time. We are at present on the wave of reaction against expectant treatment. In Paris, in the early part of this century, the student studied the natural history of disease, but the expectant plan was now a reaction in the opposite direction, not exactly toward polypharmacy, but over-active treatment.

In studying statistics of hospital administration, they seemed to be almost uniformly one sided; all facts on one side were marshalled, and those on the other unnoticed. This paper was valuable on account of giving a fair statement of the other side. As Dr. Skeer remarked, parturition was merely a physiological process, as much so as urination or defecation ; and to interfere as we all do in various ways, seems as irrational and useless as meddling with the process of defecation.

As long as a woman is doing well, would let her alone; and his own experience coincides with Dr. Lydston's. Dreaded also the officious nurse; she is dangerous in the lying-in room. Cannot remember of having lost a case of puerperal fever in private practice, but passed through an epidemic of the fever when in Bellevue Hospital, under the old system.

Cases he would fear to handle the most are those of young women not long married, boarding in luxurious style, shut up in hot rooms without exercise; an unhealthy condition of the whole system results, making parturition almost an unnatural process, just as it is a truly natural one when the mode of living has been healthy. There may be sometimes a little fever, but when we consider the process of retrogressive metamorphosis in the uterus, it is no wonder the nervous system sometimes reacts.

In these cases, generally gives ten grains blue mass followed by a saline, and found matters relieved in twenty-four hours. True puerperal fever is uncommonly met with ; he had long believed that abscess in the breast was due to mild forms of blood poisoning ;—local pyæmia in point of least resistance. We ought not to impress on the patients that the puerperal status is a dangerous one. They will oppose nature and remain childless. Meddlesome midwifery was indeed the worst kind of midwifery.

Dr. Lyons then inquired of Dr. Lydston whether the best obstetricians were now using the binder in all cases, and was answered that among the French it is recently being omitted, also, some are doing so in New York, but thought the majority, like himself, always applied a loose binder, and thought it as much a matter of policy as necessity. He would not like to condemn all injections, only perhaps those for prophylaxis. Cleanliness is essential, and for this use a vaginal injection once in twelve hours, of a quart of warm water, to which has been added a teaspoonful of compound tincture of iodine as a deodorizer and detergent, and especially good as an antiseptic and destroyer of bacteria. Also uses oakum to absorb all discharges ; of advantage on account of cheapness and cleanliness.

Dr. McCullough stated that his experience has been to always use some antiseptic in the injections, and thought the patient required less attendance.

Dr. Landis, in reply to a general question of Dr. Lydston as to experience of members in cases of puerperal fever following illegitimate births, stated that he had had many cases of illegitimate labor, but never a case of puerperal fever among them. Never, in any case, uses prophylactic measures in private practice, and in hospitals thought interference did harm. He then related a case of labor complicated by erysipelas, mentioned by Dr. Lydston, and with recovery.

Dr. Angear had had a few cases of illegitimate labor, but they were followed by no nervous disturbance, and in one other case, where woman's husband had been killed just before labor, no puerperal disturbance occurred.

Present, fifteen members and several visitors.

The Society, on motion, adjourned.

J. H. TEBBETTS, *Secretary.*

---

*July 14, 1884.*—President Angear in the chair. Dr. Holmes presented the globe which had been removed from a boy 13

years of age. The walls had been perforated with a small knife from below the center of the cornea into the sclerotic through the iris and lens.

Four months after the injury the eye had so far recovered that its general appearance might lead one to believe all danger was past. The globe, however, was tender on gentle pressure. The other eye was suffering from intolerance of light and inability to read, but for a moment.

Bisection of the organ revealed a total detachment of the retina. Inflammation of the intraocular tissues had caused an exudation of a brownish serum between the choroid and retina, separating the latter throughout its whole extent from the choroid. As the vitreous humor became absorbed, the retina, by the increase of the fluid external to it, was pressed equally towards the anterio-posterior axis of the globe. The retina thus presented the form of a partially open umbrella—the apex of the cone being at the optic nerve disk, its base being near the periphery of the iris.

An eye in this condition might suffer at any time from an attack of inflammation, which would ruin the vision of the opposite side. Hence the extirpation seemed imperative.

Dr. Holmes also exhibited two typical specimens of sarcomatous tumor of the choroid. One was a very dark melanotic tumor, situated very near the iris—in fact, encroaching upon it—and could be easily diagnosticated in its early stages as a dark tumor. The patient, a female, 45 years of age, had observed a dimness of vision five months before the extirpation. Pain had been experienced only during the last three weeks.

The other tumor was located in the fundus, near the optic nerve disk, of a lady sixty-two years of age. The patient had suffered severe pain, with attacks of inflammation, during the sixteen weeks previous to the extirpation. At this time the pupil was much dilated, the iris discolored, the lens opaque and the conjunctiva red. The fundus could not be examined by the ophthalmoscope. The tension was greatly increased. From the history of the case, it was diagnosticated as acute glaucoma, passing into a chronic form.

The examinations of the globe, however, revealed the tumor, a white-celled sarcoma, which was the cause of the secondary glaucomatous symptoms.

These two tumors are of a quite malignant character, and should always be extirpated with the whole globe as soon as discovered. Otherwise they are almost certain to invade, with rapidity, the tissues in and adjoining the orbit.

*A Case of Nerve Stretching.*— Dr. Lyons read the report of this case, prepared by Dr. Ristine, of Iowa.

A soldier, who had been wounded near the middle of the thigh, had suffered for a long period with great neuralgic pain. The operation was performed by Dr. Ristine on the sciatic and anterior crural nerves. The patient made a quite rapid recovery, and for more than four years, to the time of the report, had been free from pain.

*A Case of Intussusception.*— Dr. Skeer presented a pathological specimen, removed from the abdomen of an infant nearly six months of age, which had died with symptoms of obstruction, after an illness of eight days.

There had been great and constant pain from the commencement of the attack, with vomiting and slight tenesmus and tympanitis. The vomiting was not stercoraceous in character.

The specimen was somewhat remarkable in appearance. About an inch and a half of the ileum, nearly the whole of the cecum and appendix had passed into the colon and become constricted. The whole colon was reduced to the form of a small cord.

